# Whole-body magnetic resonance imaging (WB-MRI) for cancer screening: recommendations for use

**DOI:** 10.1007/s11547-021-01392-2

**Published:** 2021-08-02

**Authors:** Giuseppe Petralia, Fabio Zugni, Paul E. Summers, Alberto Colombo, Paola Pricolo, Luigi Grazioli, Stefano Colagrande, Andrea Giovagnoni, Anwar R. Padhani, On behalf of the Italian Working Group on Magnetic Resonance

**Affiliations:** 1 Precision Imaging and Research Unit, Department of Radiology, IEO European Institute of Oncology IRCCS, Milan, Italy; 2 Department of Oncology and Hematology, University of Milan, Milan, Italy; 3 Division of Radiology, IEO European Institute of Oncology IRCCS, Milan, Italy; 4 First Department of Radiology, Civic and University Hospital of Brescia, Brescia, Italy; 5 Department of Experimental and Clinical Biomedical Sciences, Radiodiagnostic Unit N. 2, University of Florence, Azienda Ospedaliero-Universitaria Careggi, Florence, Italy; 6 Department of Radiology, Ospedali Riuniti, Università Politecnica Delle Marche, Ancona, Italy; 7 Paul Strickland Scanner Centre, Mount Vernon Hospital, Northwood, UK

**Keywords:** Magnetic resonance imaging, Diffusion-weighted imaging, Oncology, Whole-body MRI, Cancer screening, Cancer-related syndromes

## Abstract

Whole-body magnetic resonance imaging (WB-MRI) is currently recommended for cancer screening in adult and paediatric subjects with cancer predisposition syndromes, representing a substantial aid for prolonging health and survival of these subjects with a high oncological risk. Additionally, the number of studies exploring the use of WB-MRI for cancer screening in asymptomatic subjects from the general population is growing. The primary aim of this review was to analyse the acquisition protocols found in the literature, in order to identify common sequences across published studies and to discuss the need of additional ones for specific populations. The secondary aim of this review was to provide a synthesis of current recommendations regarding the use of WB-MRI for cancer screening.

## Introduction

Whole-body MRI (WB-MRI) is a powerful imaging modality for the detection and characterization of pathologies in multiple organs, that can provide a wide anatomical coverage without exposing subjects to ionizing radiation. Improvements in scanner performance and optimization of pulse sequences have reduced acquisition times and paved the way to the adoption of WB-MRI in several clinical contexts [1]. Currently, the utility of WB-MRI in oncology is well recognized [2–4], the technical aspects of the imaging protocols, image interpretation and structured reporting have been widely discussed [5], and guidelines developed for application in advanced prostate cancer [6] and multiple myeloma [7]. In addition, WB-MRI has validated application in diagnosis and follow-up of some diseases, such as multiple myeloma [8], high-risk prostate cancer [9, 10] and melanoma [2, 3].

The high sensitivity and specificity of WB-MRI for the detection malignant tumours was demonstrated by Li et al. [11] in a meta-analysis including 1,067 cancer patients, where the diagnostic performance of WB-MRI was seen to be comparable to Positron Emission Tomography/Computed Tomography (PET/CT) for the detection of primary tumours and distant metastases. The excellent performance of MRI in the detection of bone metastases is comparable with that of PET/CT, as documented in meta-analyses published in 2014 by Shen et al. [12], in 2016 by Liu et al. [13] and in 2018 by Woo et al. [14] including 1102, 1598 and 1031 patients, respectively. The results of these studies showed pooled sensitivities of WB-MRI ranging between 0.94 and 0.97, and pooled specificities ranging between 0.94 and 0.98, making MRI the best imaging modality for the detection of bone metastases. Two multicentre trials published in 2019, investigating the use of WB-MRI for TNM staging of colorectal cancer and lung cancer [15, 16], found WB-MRI to be comparable to conventional staging pathways in terms of accuracy, while providing a reduction in staging time, number of imaging procedures and economic costs.

In the light of the good diagnostic performance of WB-MRI for tumour detection in cancer patients, several studies have been published regarding its use for cancer screening in subjects with genetic cancer predisposition syndromes, and a few papers have described its use for cancer screening in the general population, in the setting of preventive medicine (Table 2).

In this systematic review, we analyse the methodology and the results of the original research articles covering the use of WB-MRI for cancer screening in patients with cancer predisposition syndromes and in asymptomatic subjects of the general population. Firstly, we provide a synthesis of the different WB-MRI imaging protocols used for cancer screening, identifying a common core that could be used in the future studies. Secondly, we review the current recommendations and key uses of WB-MRI for cancer screening in individuals with cancer predisposition syndromes, providing also a synthesis of the current experience on its use in asymptomatic subjects of the general population.

## Study selection criteria

Medline library was used to search for eligible studies. Two separate searches were performed, the first focussed on patients with cancer predisposition syndromes and the second on asymptomatic subjects of the general population studies. Inclusion and exclusion criteria were the same for both searches.

### Inclusion criteria

- Original research articles published between 2005 and 2021.
- Details on the WB-MRI acquisition protocols must be reported in the article.
- Number of included subjects/patients and number of malignant tumours detected by WB-MRI must be reported in the article.

### Exclusion criteria

- Articles written in languages other than English.
- Review articles, research letters, posters and congress abstracts.
- Studies using multi-part MRI protocols.
- Studies using imaging techniques other than WB-MRI.

### Patients with cancer predisposition syndromes

The following search criteria were entered: (((whole-body imaging[MeSH Terms]) AND (magnetic resonance imaging[Mesh Terms])) OR (whole-body magnetic resonance imaging[Title/Abstract]) OR (whole-body magnetic resonance imaging[Title/Abstract]) OR (whole-body MRI[Title/Abstract]) OR (whole-body MRI[Title/Abstract])) AND ((genetic predisposition[MeSH Terms]) OR (paediatric[MeSH Terms]) OR (child[MeSH Terms]) OR (infant[MeSH Terms])) AND ((Neoplasm[MeSH Terms]) OR (Oncology[MeSH Terms])). The search yielded 140 results, out of which 130 were discarded after reviewing the titles and abstracts, and 10 were selected according to our inclusion and exclusion criteria. Since one study comprised the population of a previous study published by the same group of authors [17], we chose to include only the most recent one. Nine studies were finally reviewed [18–26] (Table 1).

**Table 1:** Reviewed studies in subjects with cancer predisposition syndromes

Authors	Study characteristics				Core protocol										
	Year	No. of subjects	Study population	Country	Magnet strength	Average scanning time (min)	Contrast adm	T1 W sequence	T1 W axial	T1 W coronal	T2 W axial	T2 W coronal	T2 W FS axial	T2 W FS coronal	DWI
Jasperson [18]	2013	34	Surveillance HPP (paediatric and adult)	USA	–	–	Conditional				Skull base-pelvis	Skull base-pelvis			
Anupindi [19]	2015	24	Surveillance LFS, VHL, MHE, RTS (paediatric)	USA	1.5, 3.0	72	N	TSE		Vertex-ankle	Chest-abdomen	Vertex-ankle	Head-neck-lower limbs	Vertex-ankle	
Villani [20]	2016	59	Surveillance LFS (paediatric and adult)	CAN	–	15	N							Vertex-toe	
Mai [22]	2017	116	Surveillance LFS (paediatric and adult)	USA	1.5, 3.0	60	Y	GRE	Chest-abdomen-pelvis	Vertex-toe			Vertex-toe	Vertex-toe	
Bojadzieva [23]	2017	53	Surveillance LFS (paediatric and adult)	USA	1.5	–	Y	GRE	Vertex-toe	Vertex-toe	Vertex-toe	Vertex-toe			Vertex-toe
Saya [21]	2017	44	Surveillance LFS (adult)	UK	1.5	60	N	GRE	Vertex-toe	Vertex-toe			Vertex-toe		Vertex-toe
O'Neill [24]	2017	17	Surveillance LFS (paediatric)	USA	3.0	75	N	TSE		Vertex-knee	Chest		Vertex-knee	Vertex-knee	Chest-abdomen-pelvis
Paixao [25]	2018	59	Surveillance LFS (paediatric and adult)	BRA	1.5	35	N	TSE	Vertex-toe					Vertex-toe	Vertex-toe
Friedman [26]	2020	47	Surveillance hereditary retinoblastoma (paediatric)	USA	1.5	90	N	Unknown	Vertex-toe	Whole-body	Whole-body		Vertex-toe		Vertex-toe

Authors	Study characteristics				Extensions				Patients with suspicious cancers detected by WB-MRI (%)		Confirmed by histology
	Year	No. of subjects	Study population	Country	Dedicated brain MRI	Spine	Lower limbs	Other			
Jasperson [18]	2013	34	Surveillance HPP (paediatric and adult)	USA				Targeted on suspicious findings	6	17.6	Y
Anupindi [19]	2015	24	Surveillance LFS, VHL, MHE, RTS (paediatric)	USA		T2	Yes		1	4.2	Y
Villani [20]	2016	59	Surveillance LFS (paediatric and adult)	CAN	Yes		Yes	Breast MRI	8	13.6	Y
Mai [22]	2017	116	Surveillance LFS (paediatric and adult)	USA	Yes		Yes	Breast MRI	5	4.3	Y
Bojadzieva [23]	2017	53	Surveillance LFS (paediatric and adult)	USA	Yes	T1 post C	Yes		7	13.2	Y
Saya [21]	2017	44	Surveillance LFS (adult)	UK			Yes		4	9.1	Y
O'Neill [24]	2017	17	Surveillance LFS (paediatric)	USA		T2 FS	Yes	Brain FLAIR	2	11.8	N
Paixao [25]	2018	59	Surveillance LFS (paediatric and adult)	BRA			Yes		3	5.1	Y
Friedman [26]	2020	47	Surveillance hereditary retinoblastoma (paediatric)	USA		T1, STIR	Yes		2	4.3	Y

*W* weighted, *FS* fat saturated, *Y* yes, *N* no, *GRE* gradient recalled echo, *TSE* turbo spin echo, *DWI* diffusion-weighted imaging, *STIR* short tau inversion recovery, *LFS* Li Fraumeni Syndrome, *HPP* hereditary paraganglioma pheocromocytoma syndrome, *NF* neurofibromatosis, *MHE* Multiple Hereditary Exostoses, *VHL* Von Hippel Lindau Syndrome

### Asymptomatic subjects of the general population

The following search criteria were entered: (((whole body[MeSH Terms]) AND (magnetic resonance imaging[MeSH Terms])) OR ((whole body[Title/Abstract]) OR (whole-body[Title/Abstract]) OR (total body[Title/Abstract]) OR (total-body[Title/Abstract]) OR (full body[Title/Abstract]) OR (full-body[Title/Abstract])) AND ((MRI[Title/Abstract]) OR (magnetic resonance imaging[Title/Abstract]))) AND ((early diagnosis[MeSH Terms]) OR (mass screening[MeSH Terms]) OR (population surveillance[MeSH Terms]) OR (early detection of cancer[MeSH Terms]) OR (screening[Title/Abstract])) AND ((subjects[MeSH Terms]) OR (general population[MeSH Terms]) OR (humans[MeSH Terms]) OR (subjects[Title/Abstract]) OR (general population[Title/Abstract]) OR (humans[Title/Abstract])). The search yielded 276 results, out of which 265 were discarded after reviewing the titles and abstracts, and 11 were selected according to our inclusion and exclusion criteria. Two additional studies were later found during the review process by cross-checking of citations [27, 28] and included in the analysis. We noticed, however, that one of them [24] was an update with a larger population of a pilot study previously selected [25]. Therefore, we kept the updated study with the larger population [24] and withdrew from the pool of analysed studies the pilot study [29]. Twelve articles were finally reviewed [21, 27, 28, 30–38] (Table 2). Of note, an article included both subjects with a cancer predisposition syndrome and a cohort of asymptomatic subjects of the general population [21], and, for this reason, was included in both the searches performed.

**Table 2:** Reviewed studies in asymptomatic subjects of the general population

Authors	Study characteristics				Core protocol										
	Year	No. of subjects	Study population	Country	Magnet strength	Average scanning time (min)	Contrast adm	T1 W sequence	T1 W axial	T1 W coronal	T2 W axial	T2 W coronal	T2 W FS axial	T2 W FS coronal	DWI
Goehde [32]	2005	298	General population	GER	1.5	50	Y	GRE		Vertex-toe	Chest				
Baumgart [33]	2007	1007	General population	GER	1.5	60	Y	GRE	Chest	Vertex-toe	Chest, pelvis				
Lo [34]	2008	132	General population	HKG	3.0	33	N	GRE	Chest-pelvis	Vertex-pelvis	Vertex-pelvis				
Takahara [35]	2008	10	General population	NED	1.5	38	N	GRE		Vertex-pelvis		Vertex-pelvis			Vertex-pelvis
Hegenscheid [26]	2013	2500	General population	GER	1.5	45	Conditional	GRE	Vertex-abdomen	Vertex-toe	Chest-abdomen			Vertex-toe	Abdomen-pelvis
Cieszanowski [36]	2014	666	General population	PLN	1.5	50	N	GRE	Chest-pelvis		Vertex-pelvis			Vertex-toe	
Tarnoki [37]	2015	22	General population	GER	3.0	–	Y	TSE GRE		Vertex-toe				Vertex-toe	Vertex-pelvis
Ulus [38]	2016	116	General population	TUR	1.5	30	N	TSE	Abdomen		Vertex-pelvis			Vertex-toe	abdomen
Saya [21]	2017	44	General population (control subjects)	UK	1.5	60	N	GRE	Vertex-toe	Vertex-toe			Vertex-toe		Vertex-toe
Lee [30]	2018	229	General population	KOR	1.5	30	N	GRE		Vertex-toe				Vertex-toe	
Hou [28]	2020	209	General population	USA	3.0	-	N	GRE	Whole-body (vertex-pelvis)	Vertex-toe	Whole-body (vertex-pelvis)				Whole-body (vertex-pelvis)
Basar [31]	2021	576	General population	TUR	1.5–3.0	45–55	N	GRE	Chest-abdomen-pelvis		Vertex-knee	Vertex-knee		Vertex-knee	Vertex-knee

Authors	Study characteristics				Extensions				Patients with suspicious cancers detected by WB-MRI (%)		Confirmed by histology
	Year	No. of subjects	Study population	Country	Dedicated brain MRI	Spine	Lower limbs	Other			
Goehde [32]	2005	298	General population	GER	Yes		Yes	MRI colonography, cardiac MRI, MRI angiography	1	0.3	Y
Baumgart [33]	2007	1007	General population	GER	Yes		Yes	MRI colonography, cardiac MRI, MRI angiography	4	0.4	Y
Lo [34]	2008	132	General population	HKG		T2			4	3.0	Y
Takahara [35]	2008	10	General population	NED					1	10.0	Y
Hegenscheid [26]	2013	2500	General population	GER	Yes	T1, T2	Yes	Breast MRI, cardiac MRI, MRI angiography	62	2.5	N
Cieszanowski [36]	2014	666	General population	PLN		T2	Yes		7	1.1	Y
Tarnoki [37]	2015	22	General population	GER			Yes	MRI angiography	1	4.5	N
Ulus [38]	2016	116	General population	TUR			Yes		2	1.7	Y
Saya [21]	2017	44	General population (control subjects)	UK			Yes		0	0	Y
Lee [30]	2018	229	General population	KOR			Yes		2	0.9	Y
Hou [28]	2020	209	General population	USA					20	1.7	Y
Basar [31]	2021	576	General population	TUR	Yes	T2		Prostate MRI (T2, DWI)	15	2.6	Y

*W* weighted, *FS* fat saturated,  yes, *N* no, *GRE* gradient recalled echo, *TSE* turbo spin echo, *DWI* diffusion-weighted imaging, *STIR* short tau inversion recovery

## Imaging acquisition protocol

When WB-MRI is performed for cancer detection, the acquisition protocol should be tailored to the specific population being evaluated. After the discussion of the technical aspects of WB-MRI examinations in the study included in this review, we propose a common “core protocol” for WB-MRI examinations, and then describe the imaging extensions to the core protocol that address the needs of specific populations.

### Hardware

The studies included in this review cover WB-MRI examinations performed on both 1.5 Tesla (T) and 3 T MRI scanners (Tables 1, 2). Generally, it is preferable for patients with non-removable metallic prostheses to undergo scanning on 1.5 T systems to limit susceptibility artefacts and image distortion. For imaging at 3 T, scanners should feature multi-transmit technology to provide homogeneous images. Irrespective of field strength, parallel imaging is useful for keeping acquisition times down, while multi-station planning and acquisition are essential to allowing whole-body coverage. The coils required include head and neck, spine and enough anterior array coils to provide the desired anatomical coverage. In general, denser coil arrays permit greater acceleration and thus faster imaging [39, 40], but as yet specific recommendations are lacking regarding the number of coil elements required for optimal image quality. Current scanners allow the core protocol images to be obtained in clinically acceptable times, ranging from 30 to 40 min for head to pelvis, and up to 60 min when including lower limbs.

### Use of contrast agents

Contrast agents (CA) were administered only in three out of the nine studies performed in patients with cancer predisposition syndromes.

In two studies [22, 23] performed in patients with Li–Fraumeni Syndrome (LFS), the protocol included dedicated brain assessment in the same sitting of WB-MRI, therefore requiring CA administration. Although not needed for the core WB-MRI protocol, CA should be administered when additional dedicated brain evaluations are performed in the same sitting. This may apply, for example, to patients with LFS [41], neurofibromatosis (NF) [42] and Constitutional Mismatch Repair Deficiency Syndrome (CMMR-D) [43].

In the remaining study by Jasperson et al. [18], targeted post-contrast acquisitions were performed in patients with hereditary paraganglioma pheocromocytoma syndrome (HPP), when the unenhanced T2 weighted images showed abnormal findings, suspicious for pheochromocytoma, paraganglioma or other succinate dehydrogenase (SDH) related tumours, such as GIST, oncocytoma or renal cancer.

In the majority of studies on asymptomatic subjects of the general population included in this review (8 out of the 12), no CA were administered, as considered not necessary. In the remaining 4 studies, CA were administered for the only purpose of performing additional dedicated MRI studies in the same sitting, such as breast MRI, MR colonography, MRI angiography and cardiac MRI [27, 32, 33, 37]. Of note, all the malignant tumours reported in the largest of these four studies [27] with CA administration, including 2500 subjects, were detected on whole-body unenhanced MR images, with the exception of a myocardial tumour of unknown histotype found in the targeted contrast-enhanced cardiac MRI.

Medical and public concerns related to long-term gadolinium deposition in the brain [44] and possible adverse effects in subjects with undisclosed acute kidney injury, including nephrogenic systemic fibrosis [45], further discourage the use of injected CA. Finally, the discomfort related to intravenous puncture may discourage some subjects to undergo the examination.

Therefore, the routine use of CA when performing WB-MRI for cancer screening in asymptomatic subjects of the general population is strongly discouraged.

### Morphologic images

Most of the reviewed articles made use of a combination of whole-body T1-weighted and T2-weighted images. Most of the studies that featured whole-body T1-weighted imaging (14 of 19 studies) made use of gradient echo (GRE) sequences for the T1-weighted images (Tables 1, 2). The main advantages of GRE sequences are shorter acquisition times compared to fast spin-echo sequences, and the possibility of acquiring 3D image volumes. A distinctive additional advantage of GRE acquisition is the ability to use the Dixon method to produce multiple image contrasts, including in-phase, opposed-phase, fat-only and water-only images (equivalent of fat-suppressed images) within a single acquisition. A particular feature of the Dixon technique is the ability to calculate relative fat-fraction (rF%) maps by dividing the signal intensity of fat-only images by the sum of fat-only and water-only images, via the formula: “100 × (F∕(F + W))”. Fat-fraction maps enable a quantitative representation of the content of fat within tissues and represent a further diagnostic aid (at no expenses of acquisition times) for the detection and characterization of lesions in the bone marrow, and other organs (liver, adrenals, ovary) by discriminating malignancies from benign lesions based on their fat content [46, 47].

Whole-body T2-weighted images were obtained in 19 of the 21 studies reviewed (Tables 1, 2). In five of these studies, the T2 weighted images were acquired without fat-suppression [18, 23, 28, 34, 35], while in nine [20–22, 24, 25, 27, 30, 37] only fat-suppressed T2 weighted images were acquired. In the remaining five studies, T2-weighted images were acquired both with and without fat-suppression [17, 19, 31, 36, 38]. Half-Fourier single-shot turbo spin-echo (HASTE) was the sequence commonly used without fat-suppression. Short tau inversion recovery (STIR) with turbo spin-echo acquisition was used in 11 of the 14 studies in which fat-suppressed whole-body T2 weighted images were acquired [17, 19, 20, 22, 25, 27, 30, 31, 36–38]. The use of STIR was initially motivated by its uniform fat suppression over large fields-of-view and higher sensitivity for bone oedema and skeletal lesions, but the role of STIR in bone evaluations has largely been ceded to diffusion-weighted imaging (DWI), which has seen increasing use in oncologic studies over time. STIR sequences remain useful in WB-MRI for the evaluation of specific regions, such as the spine, thanks to their superior spatial resolution compared to DWI, and for the assessment of peripheral nerve sheath tumours in patients with neurofibromatosis [48].

### Diffusion-weighted imaging

Over the last decade, the role of DWI in cancer imaging has increased tremendously, because its high sensitivity and specificity, that has increased the diagnostic performance of MRI studies and reduced the need for contrast medium administration. The advantages observed across multiple anatomical regions, including upper abdomen [49], genitourinary tract [50] and bone [14], derive from the qualitative analysis of images, while no added value in clinical routine has been demonstrated for quantitative measurements of apparent diffusion coefficient (ADC) in the setting of lesion detection and characterization. The use of ADC cut-off values in clinical routine is limited by variability in ADC values, depending heavily on imaging parameters and specification of the MR unit (e.g. magnetic field strength, gradients, coils), as well as on additional factors, including artefacts (e.g. metal implants, air-tissue interfaces, motion) [51] or age and gender in bone marrow, partly related to age related changes in bone marrow fat content [52].

In addition, the added value of performing DWI has not been formally evaluated in WB-MRI examinations performed for cancer screening. Despite being a mainstay in current oncologic WB-MRI protocols [6, 7], DWI was used for whole-body evaluation in only 10 of the 21 studies analysed, and was used for the evaluation of the abdomen in further two studies. In older studies, the exclusion of DWI might have been motivated by the previously inadequate quality of DWI on most scanners [32–34]. In other studies, especially in those performed for annual surveillance of subjects with cancer predisposition syndromes, it could have been justified by the need to keep examination times as short as possible, especially when children are being screened [19, 20, 22].

Besides being highly sensitive for tissues with a high cellularity, DWI reduces the need for contrast administration for lesion characterization and is therefore intrinsically attractive for cancer screening studies. Single-shot echo planar imaging (SSH-EPI) sequences are recommended, with the acquisition of at least two b values being necessary to allow the calculation of ADC [53]. While the highest b value should range between 800 and 1000 s/mm^2^, the lowest should not be lower than 50 s/mm^2^, in order to reduce perfusion-related signals [54]. STIR preparation pulses are recommended in order to achieve uniform fat suppression over large fields-of-view. The DWI images should be acquired with anterior to posterior phase encoding during free breathing, and with multiple averages in order to reduce motion artefacts and increase SNR [6, 7, 54]. Radial maximum intensity projections, reconstructed from high b value images, are commonly used for navigation and at-a-glance detection of hyper-intense lesions across the body [6, 7].

### Anatomical coverage

All WB-MRI protocols covered at least from head to pelvis. This anatomical extent should be considered as the minimum coverage for WB-MRI. Lower limbs were covered by the WB-MRI protocol in eight out of nine studies performed in cancer predisposed populations, and in eight studies out of the 12 performed in the general population. Interestingly, no malignant tumours were diagnosed in the lower limbs across in the general population, despite over 4800 subjects being scanned [55]. We suggest, therefore, that the lower limbs evaluation should be excluded from the core WB-MRI protocol, as it is unlikely to increase diagnostic yields, in spite of a considerable increase in acquisition times. This body region must be evaluated only in those subjects who present with a higher risk of cancer in the long bones or in soft tissues.

The choice of acquiring morphological images in the axial or coronal plane is still subject to radiologists’ preferences. The coronal plane was favoured in early WB-MRI studies, when examinations were more time consuming, because it requires fewer slices acquired antero-posteriorly than axial imaging to cover the entire body volume, and thereby reduce scanning times. The advantages of axial studies include the possibility of matching morphological images to DWI, as well as comparing MRI with other cross-sectional imaging modalities such as CT. With the introduction of parallel imaging, the optimization of software solutions for sequence planning, and the growing clinical demand for WB-MRI in oncologic patients, radiologists are increasingly adopting the axial orientation for WB-MRI studies.

### WB-MRI “core Protocol” and extensions

**Fig. 1:**
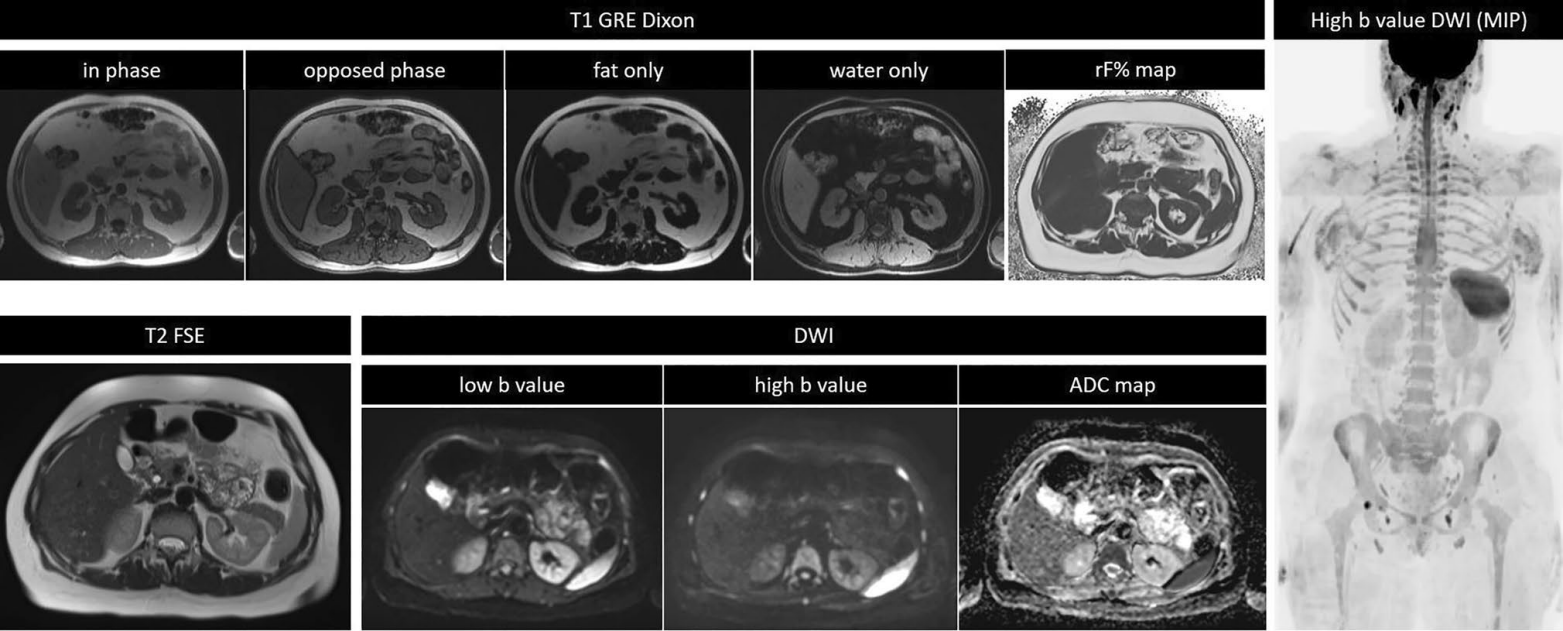
Core protocol. This image summarizes the main pulse sequences included in the WB-MRI protocol for cancer screening. The study should cover from head to pelvis. T1 weighted images are acquired with a gradient recalled echo (GRE) sequence, preferably with Dixon technique, to obtain four different sets of images (in-phase, opposed-phase, water-only, fat-only) and to allow the calculation of the relative fat-fraction (rF%) map. T2 images are acquired with a single-shot fast spin echo (FSE) sequence. Diffusion-weighted images (DWI) should be acquired with at least two b values in order to obtain the corresponding apparent diffusion coefficient (ADC) map. Maximum intensity projections (MIP) should be reconstructed from the highest b value DWI images

Considering the above discussion, we propose a “core protocol” for WB-MRI composed of T1 weighted GRE sequences together with T2 weighted TSE sequences and DWI, as described in Table 3 and in Fig. 1. Anatomical coverage, field of view and slice thickness should be homogeneous across the sequences, in order to facilitate image correlation and interpretation. If available, automatic image composing should be enabled on the scanner during acquisition, in order to produce stacks for each type of image generated without the need for manual intervention.

**Table 3:** Core imaging protocol for WB-MRI

Sequence	Anatomical coverage	In-line reconstructions	Post-processing
T1-weighted GRE • Dixon technique	Vertex to pelvis • Multiple stations • Contiguous 5 mm slices	Unified stacks • In-phase • Opposed-phase • Water-only • Fat-only	Relative fat-fraction map
T2-weighted TSE • No fat suppression	Vertex to pelvis • Multiple stations • Contiguous 5 mm slices	Unified stack	
DWI • b50 and b800-1000 s/mm^2^ • STIR fat suppression	Vertex to pelvis • Multiple stations • Contiguous 5 mm slices	Unified stacks • Low b value • High b value • ADC map	MIP of highest b-value • Rotation around craniocaudal axis at 3° steps

*GRE* gradient recalled echo, *TSE* turbo spin echo, *DWI* diffusion-weighted imaging, *STIR* short tau inversion recovery, *ADC* apparent diffusion coefficient, *MIP* maximum intensity projection

**Fig. 2:**
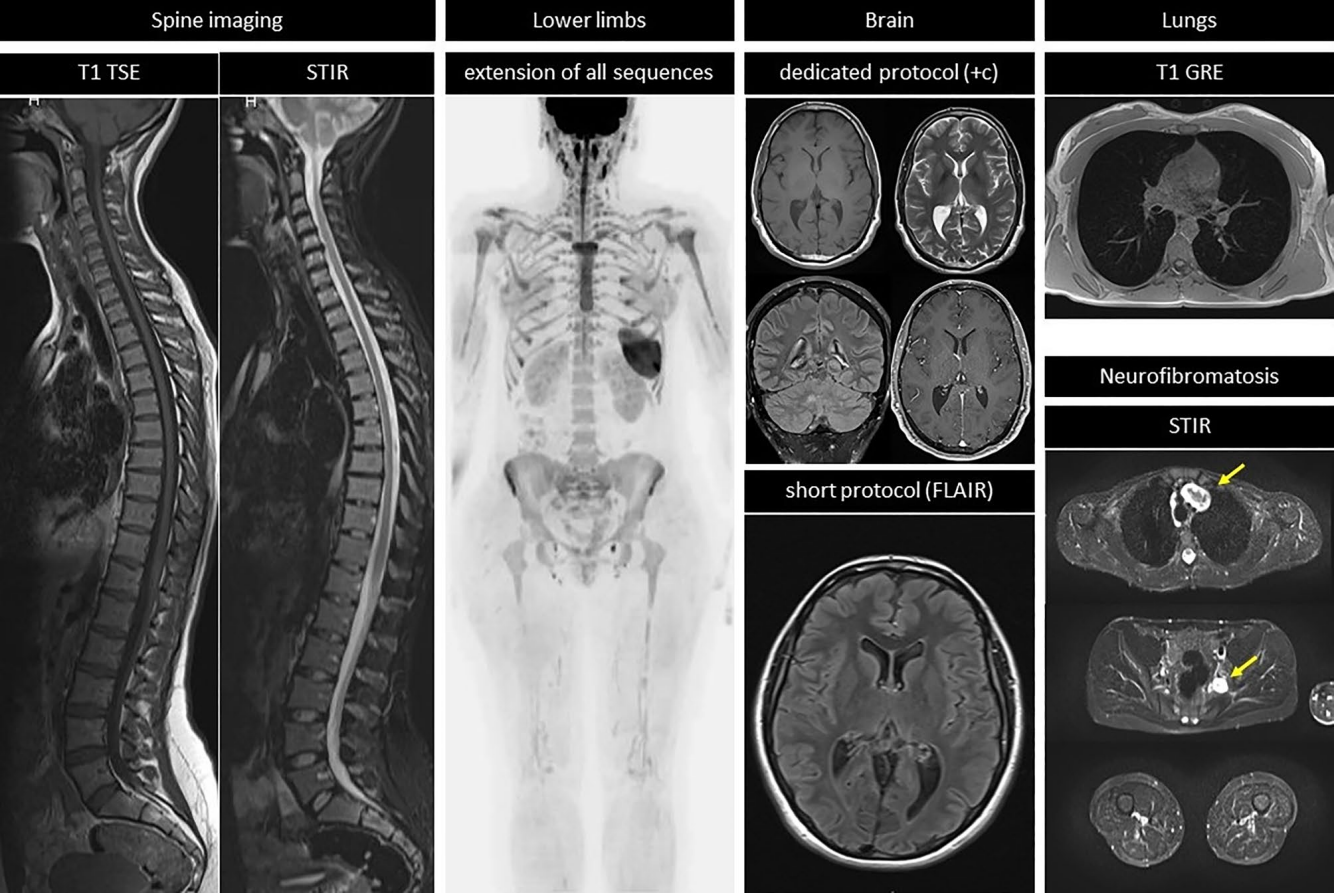
Extensions to the core protocol. This picture summarizes possible complements to the core protocol. When assessment of the spine is required, sagittal short tau inversion recovery (STIR) is used. In subjects with a high risk of vertebral tumours, a sagittal T1 weighted turbo spin echo (TSE) sequence can be additionally performed. When the WB-MRI protocol includes the lower limbs, such as in subjects with Li Fraumeni syndrome, all sequences in the core protocol are extended to the feet. When there is an increased risk of central nervous system (CNS) tumours, a dedicated brain sub-protocol is performed, with multiple sequences and with contrast administration (+ c). In subjects with a low risk of CNS tumours, the assessment of the brain can be improved with a short brain protocol, including fluid attenuated inversion recovery (FLAIR) sequences. Single breath-hold T1 weighted gradient recalled echo (GRE) sequences can be performed for the assessment of the lungs. In subjects with neurofibromatosis, the STIR sequence should be performed in either the axial or the coronal plane, covering from neck to feet, for facilitating the detection of peripheral nerve sheath tumours (arrows)

Extensions to the core protocol should be implemented in high-risk populations (Table 4). These may include sagittal imaging of the whole spine, dedicated brain imaging, MRI mammography, and coverage of the lower limbs. Of note, the administration of CA may be required in subjects with cancer predisposition syndromes (e.g. Li–Fraumeni syndrome) for better assessment of specific body regions (e.g. brain) (Fig. 2).

**Table 4:** Extensions to the core protocol

Sequence	Anatomical coverage	Indication
Spine imaging • T1-weighted TSE • T2-weighted STIR	Sagittal Contiguous slices, 4–5 mm	LFS, HHP
Lower limbs • T1-weighted GRE • T2-weighted TSE • DWI	Extend coverage to feet	LFS, NF, MHE
Dedicated brain evaluation • Multiple sequences • Contrast administration		LFS, CMMR-D, NF
Short brain evaluation • T2-weighted FLAIR	Axial Contiguous 4-5 mm slices	Patients not undergoing dedicated brain examinations
Lung evaluation • T1-weighted GRE		
Detection of neurofibromas • T2-weighted STIR	Vertex to feet Axial or coronal Contiguous 4-5 mm slices	NF

*GRE* gradient recalled echo, *TSE* turbo spin echo, *DWI* diffusion-weighted imaging, *STIR* short tau inversion recovery, *FLAIR* Fluid Attenuated Inversion Recovery, *LFS* Li Fraumeni Syndrome, *HPP* hereditary paraganglioma pheocromocytoma syndrome, *NF* neurofibromatosis, *MHE* Multiple Hereditary Exostoses, *VHL* Von Hippel Lindau Syndrome, *CMMR-D* Constitutional Mismatch Repair Deficiency Syndrome

### Structured reporting

None of the studies included in this review described the use of a structured reporting template. The standardisation of WB-MRI reports in the setting of cancer screening, as pointed out by Greer in a review article published in 2017 [56], might help mitigating the “inherent interpretive variability” that occurs also among expert readers. It is now clear that the lack of a standardisation in WB-MRI reporting impairs prospective data collection, which results into a limited comparability of the results of published studies. Some efforts towards the standardisation of data collection can be found in the articles included in this review. In 14 out of 21 studies, the authors used a categorical system for abnormal WB-MRI findings, to classify them on the basis of their oncological relevance (e.g. benign vs indeterminate vs clearly malignant). The categorical systems reported showed, however, a high heterogeneity, with four studies using a binary system [18, 25, 32, 34], four studies a three-category system [23, 30, 36, 37], three a four-category system [19, 27, 31], one a five-category system [24]; one last study used a six-category system for both patients with cancer predisposition syndromes and asymptomatic subjects of the general population [21]. The recently published Oncologically Relevant Findings Reporting and Data System (ONCO-RADS) guidelines [57] represent a comprehensive effort towards the standardisation of WB-MRI reporting in the setting of cancer screening. These guidelines provide a template for structured reporting, as well as a five-category classification system for data collection and a systematic approach for the communication and management of abnormal findings.

## Subjects with cancer predisposition syndromes

### Li–Fraumeni syndrome (LFS)

**Fig. 3:**
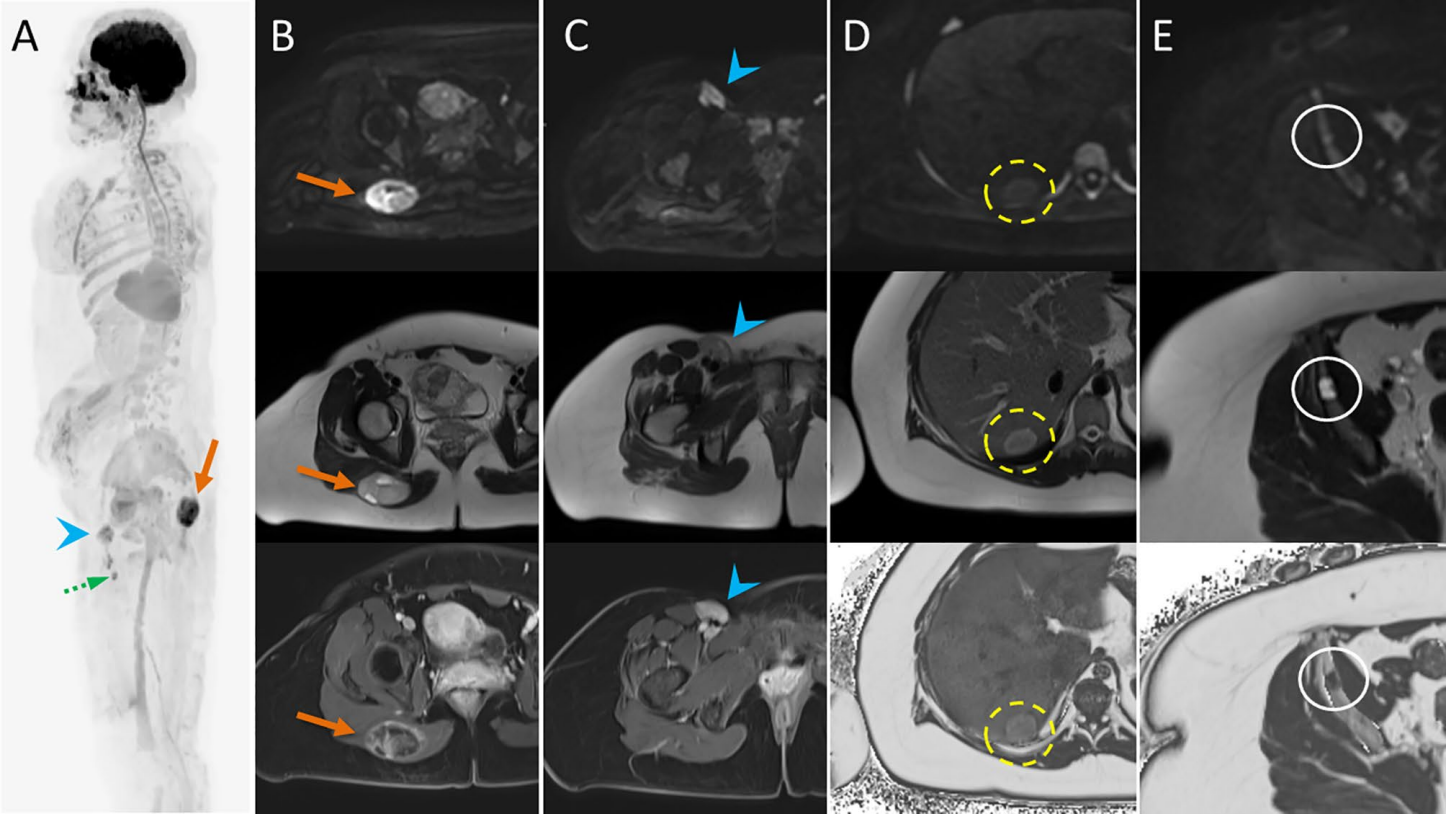
Case example. Images of a 29-year-old woman with Li-Fraumeni syndrome with prior history of giant cell fibroblastoma of the groin (13 years before), Paget disease of the right breast and grade-3 ductal intraepithelial neoplasia (DIN-3) of the right breast (8 years before). The patient underwent a first screening with WB-MRI, which revealed multiple abnormal findings. The high b-value maximum intensity projection displayed in lateral view with inverted grayscale (A) revealed three hyper-intense lesions in the pelvis. Firstly, a 8-cm mass highly suspicious for cancer was detected in the right gluteus (arrow in A and B), showing hyper intense appearance in high b-value images (top row, A and B), cystic areas in T2 weighted images (middle row, A and B) and irregular contrast enhancement in delayed post-contrast T1 weighted (W) Dixon images (bottom row, A and B). Secondly, a solid lesion with irregular shape was seen adjacent to the right femoral vessels (arrowhead in A and C), with hyper-intense appearance in high b-value images, heterogeneous signal in T2 W images and strong enhancement in post-contrast T1 W Dixon images (A and C, top, middle and bottom row, respectively). The finding was reported as strongly suspicious for local recurrence of fibroblastoma. Thirdly, an enlarged femoral lymph node was seen in the right thigh (dashed arrowhead in A). The patient underwent surgical resection of the suspicious lesion in the gluteus and dissection of the right groin, with histopathological diagnosis of high-grade sarcoma of the gluteus and local recurrence of fibroblastoma. Metastasis from high-grade sarcoma was diagnosed in the enlarged femoral lymph node. Two other focal lesions were detected, which were not visible in high b-value images (D and E, top row). In D, a solid, rounded lesion was seen the VII segment of the liver, with high signal intensity in T2 W images (D and E, middle row) and evidence of intralesional fat in the relative fat-fraction (rF%) map (D, bottom row). The lesion showed similar contrast enhancement compared to surrounding parenchyma in T1 W Dixon images (not shown). Although not suspicious for malignant cancer, the lesion was not visible in a prior MRI study; therefore, a percutaneous liver biopsy was performed, with benign findings suggestive of chronic inflammation, macrovescicular steatosis and ductal hyperplasia. Follow-up at seven months distance showed stable findings. In E, a lobulated bone lesion was seen in the right ilium, with minimal remodelling of the cortical bone and hyper-intense content in T2 W images (middle row), and intralesional fat content below 5% in the rF% map. The lesion was not visible in a prior MR study, and in the suspect of malignancy, a percutaneous biopsy was performed, which revealed sparse foci of epithelial tumoral cells, with immunohistochemical findings compatible with metastasis from occult breast cancer. Subsequent mammography and ultrasonography revealed no abnormal breast lesions and the patient is at present under strict follow-up

LFS is a rare, hereditary, autosomal dominant disorder, in which germline mutations lead to a functional inactivation of the TP53 tumour suppressor gene. As a result, the cumulative cancer incidence in subjects with LFS is markedly increased compared to the general population, reaching 50% in the third and fourth decade in females and males, respectively, while cancer prevalence approaches 100% in female subjects after the sixth decade [58]. Common malignant cancers in LFS include sarcoma, breast cancer, brain tumours, leukaemia, and adrenocortical carcinoma [20] (Fig. 3).

Seven studies published between 2015 and 2017 examined the use of WB-MRI for cancer surveillance in LFS subjects [19–25]. In these studies, the patients were participating in surveillance protocols that followed the principle of avoiding ionizing radiation exposure, and alongside other examinations, included WB-MRI with imaging protocols as reported in Table 1.

The National Cancer Institute studied 116 subjects with a germline TP53 pathogenic variant, mean age 37.6 years (range 3–68 years), with WB-MRI (without DWI) along with dedicated brain and breast MRI [22]. The presence of cancer was disclosed in 4.3% of cases. Interestingly, most prevalent cancers were diagnosed at an early stage: 2 stage I lung adenocarcinomas, 1 intermediate-grade osteosarcoma, 1 low-grade spindle cell sarcoma, and a WHO grade II brain astrocytoma.

Preliminary results from the UK SIGNIFY study on TP53 mutation carriers showed the potential of WB-MRI for baseline surveillance of individuals with LFS. Four out of 44 subjects (9.09%, median age 37 years, range 19–58) were found to have five malignancies [21].

Similar observations resulted from a cohort study in patients with LFS conducted by Bojadzieva et al. in which WB-MRI identified three paediatric low-grade gliomas, one recurrent abdominal wall sarcoma, one thyroid papillary carcinoma, one soft-tissue liposarcoma and one metastatic groin sarcoma with cancer rate of 13% [23].

A study by Anupindi et al., involving WB-MRI cancer screening in 24 paediatric subjects (mean age 11.2, range 2.1–18.2) with LFS and other cancer predisposition syndromes, observed one asymptomatic papillary cancer, resulting in a cancer rate of 4.16% [19]. Additionally, the authors pointed out that WB-MRI examinations performed in institutions with limited experience in cancer screening would likely benefit from centralized image review in institutions with a high volume of LFS patients.

A prospective observational study by Villani et al. on 89 individuals with LFS, observed a significant difference in five-years survival rates (88.8% and 59.6%, respectively) between the surveillance group (*N* = 59) undergoing WB-MRI and the non-surveillance group (*N* = 30). Over the course of the study, cancer emerged in 25.4% of the surveillance group [20].

A meta-analysis by Ballinger et al. showed a cancer prevalence of 7% among 578 subjects with LFS who underwent baseline staging with WB-MRI [59].

These data point to the usefulness of WB-MRI in the surveillance of LFS subjects and led to the publication of a guideline by the MD Anderson Cancer Centre, recommending annual WB-MRI for cancer screening in paediatric subjects with TP53 germline mutation [5]. Recently, the National Comprehensive Cancer Network (NCCN) and the American Association for Cancer Research (AACR) have issued recommendations in which WB-MRI along with annual brain MRI with CA were considered as the cancer screening technique of choice in the management of adult and paediatric subjects with LFS, with the addition of breast MRI in women [60, 61].

### Hereditary paraganglioma–pheocromocytoma syndromes (HPP)

HPP are genetic disorders characterized by tumours that originate from the neural crest. The genes responsible for HPP syndromes collectively include the SDHx genes and a group of multiple nuclear genes encoding subunits of the succinate dehydrogenase (SDH) enzyme [62]. Subjects with HPP syndromes can also develop RCC, gastrointestinal stromal tumours, pituitary adenomas, and other rare tumour types.

The prevalence of HPP syndromes is very low in the general population, and patients are typically diagnosed in their third decade [48]. HPP surveillance protocols have adopted WB-MRI with excellent diagnostic performances. In a study conducted by Jasperson et al. on 37 patients with HPP, WB-MRI showed a higher sensitivity (87.5%) for SDH-related tumours than biochemical testing (37.1%) [18]. In order to decrease morbidity and mortality from HPP-related tumours, the American Association for Cancer Research recommends biennial screening with WB-MRI for subjects with HPP syndrome older than 6/8 years of age [63].

### Constitutional mismatch repair deficiency (CMMR-D)

The CMMR-D syndrome is a rare disease caused by a homozygous mutation of the mismatch repair (MMR) gene system. CMMR-D leads to a higher risk of high-grade central nervous system neoplasms, non-Hodgkin lymphoma and acute lymphoblastic leukaemia during childhood, and colorectal and haematological neoplasms during adolescence and early adulthood. An annual WB-MRI examination in conjunction with dedicated brain MRI has been recommended for subjects with CMMR-D from 6 to 8 years of age, in a consensus statement by the Care for CMMRD Consortium and the International Biallelic Mismatch Repair Deficiency Consortium [43].

### Neurofibromatosis (NF)

Neurofibromatosis is a neuro-cutaneous genetic disorder characterized by the development of tumours throughout the nervous system. Most NF diagnoses occur during childhood or early adulthood, and for this reason a radiation-free screening imaging technique is highly desirable [64]. WB-MRI has proven its efficacy in detecting number, volume, and distribution of the three main clinical manifestations of neurofibromatosis: neurofibromatosis type 1 (NF1), neurofibromatosis type 2 (NF2), and schwannomatosis (SWN). In a study conducted by Plotkin et al. on 247 subjects with NF1, NF2 and SWN who underwent unenhanced WB-MRI without DWI, 1286 neurofibromas were found, involving 59% of the cases (145 subjects) [65].

Despite the aforementioned evidence, WB-MRI for the assessment and surveillance in NF patients is not included in current guidelines. The “Response Evaluation in Neurofibromatosis and Schwannomatosis International Collaboration” (REiNS) has, however, provided recommendations on image acquisition and analysis methods to enable WB-MRI as an endpoint in NF clinical trials.

Subjects with NF are at increased risk of developing a rare type of sarcoma termed malignant peripheral nerve sheath tumour (MPNST) during their lifetime, with cumulative risk of 8–13% [66]. As reported by Cashen et al., subjects who receive a timely diagnosis of MPNST can undergo curative treatments, with an overall survival rate of 84% [67]. For this reason, the NCCN has encouraged the development of new guidelines for the detection of MPNST, emphasizing the role of advanced imaging modalities such as WB-MRI for this task [68].

### Von Hippel–Lindau syndrome (VHL)

VHL syndrome is an autosomal dominant disorder caused by germline mutations or deletions in a tumour suppressor gene mapped to human chromosome 3p25 [69]. It manifests with hyper-vascular tumours arising in the central nervous system and in the upper abdomen. RCC is the most prevalent malignant histotype, occurring in 70% of individuals with VHL and is the leading cause of mortality.

Improved understanding of VHL genetics and biology has led to the introduction of early interventions, innovative treatments and screening protocols, all of which resulted in a substantially improved prognosis for this disease [69].

For these reasons, several national and international societies proposed MRI surveillance protocols for these subjects, including cerebral, inner-ear, spinal and upper abdominal evaluations. Such guidelines were published by the Danish VHL coordination group [70] and the American Association of Cancer Research (AACR) [63]. However, no formal consensus exists for the surveillance protocol of choice in VHL subjects.

In this context, the possibility of assessing multiple body regions in a one-stop-shop examination (and eventually without CA administration for extracranial cancer screening) such as WB-MRI seems particularly promising in the forthcoming development of suitable surveillance protocols for VHL patients.

### Multiple hereditary exostoses

Multiple hereditary exostoses (MHE) is an autosomal dominant hereditary disease characterized by the growth of multiple benign and symptomatic osteochondromas [71]. This genetically inherited disease is usually diagnosed during childhood and requires lifelong monitoring and treatment of tumours. Moreover, young subjects with MHE have an increased risk of developing chondrosarcoma as an adult. The transformation of MHE to malignant variants is seen in 2–4% of affected patients [72].

MRI represents the most valuable imaging modality in symptomatic MHE, because it can precisely depict soft-tissue pathology and differentiate malignant transformation. Moreover, the prevalence of MHE in young/paediatric subjects suggests that a dose-saving surveillance imaging method should be preferred for these patients. Therefore, screening with WB-MRI would be highly desirable. In a systematic review published in 2014, Sonne-Holm et al. proposed a screening protocol for patients with MHE, which would include lifelong biennial surveillance with WB-MRI among other interventions. The authors also suggested the examinations be centralized in high volume tumour departments, that would coordinate the screening and treatment interventions in these patients [73].

## Asymptomatic subjects of the general population

The encouraging results obtained in studies of WB-MRI for cancer screening in high-risk populations has generated a growing interest for the possible application of WB-MRI for cancer screening in the general population as an adjunct to standard screening tests (e.g. mammography, cervical cancer screening, faecal occult blood testing (FOBT)). Thanks to its wide anatomical coverage and its safety profile, WB-MRI provides a unique opportunity to detect malignant tumours in organs that are not targeted by current screening programmes, without exposing the patient to ionizing radiation or requiring CA injection. Several studies provide insights into this use of WB-MRI. While some of these involved healthy volunteers recruited as controls in studies investigating the role of WB-MRI in LFS [21], others were exploratory studies in preventive health programmes [28], and others still, such as the study by Hegenscheid et al., were large cohort studies involving thousands of subjects in which an exhaustive imaging protocol (including WB-MRI, Cardiac MRI, MRI angiography and MRI mammography) was used to search for a variety of pathologic findings, including cancer, in the general population [27]. In 12 of these studies, 5809 subjects were included. The image-acquisition protocols of these studies are summarized in Table 2. Heterogeneity in imaging protocols, methodology and follow-up assessments render the comparison of these studies difficult [55]. Nonetheless, all the reports provide the number of subjects in which a malignant tumour was suspected, with cancer detection rates from 0% up to 10%. This wide range is probably a consequence of the small sample sizes of many studies, and the above-mentioned technical heterogeneity. Taken together, findings suspicious for malignant cancers were reported in nearly 2.0% (119 of 5809) of the screened subjects. Unfortunately, the number of studies in which follow-up and verification of findings was performed is lower, comprising 3287 screened asymptomatic subjects, in whom there was a 1.5% overall rate of histologically confirmed malignant cancers. This rate of malignant tumours detected with WB-MRI in asymptomatic subjects of the general population should not be ignored, justifying further studies. However, critics highlight the high rate of indeterminate incidental and false‐positive findings, which can lead to unnecessary additional examinations and treatments, with potential negative psychological impact [74, 75]. Therefore, the clinical utility of WB-MRI for cancer screening in the general population remains a matter of debate.

## Conclusions

The WB-MRI protocols for cancer screening in the literature show similarities, based on which we suggest a “core protocol”, that includes T1-weighted GRE, T2-weighted TSE and DWI sequences for the evaluation of head, neck, chest, abdomen and pelvis. Additional sequences and sub-protocols can be performed as extensions to the core protocol, in order to adapt the WB-MRI examination to the specific risk profile of the population being evaluated.

The use of WB-MRI for cancer screening is recommended by current guidelines for subjects with cancer predisposition syndromes, including Li–Fraumeni syndrome, hereditary pheochromocytoma–paraganglioma syndromes and constitutional mismatch repair deficiency.
